# Induced seismicity closed-form traffic light system for actuarial decision-making during deep fluid injections

**DOI:** 10.1038/s41598-017-13585-9

**Published:** 2017-10-19

**Authors:** A. Mignan, M. Broccardo, S. Wiemer, D. Giardini

**Affiliations:** 1 Swiss Federal Institute of Technology Zurich, Institute of Geophysics, Zurich, Switzerland; 2 Swiss Competence Center for Energy Research – Supply of Electricity, Zurich, Switzerland; 3 Swiss Seismological Service, Zurich, Switzerland

## Abstract

The rise in the frequency of anthropogenic earthquakes due to deep fluid injections is posing serious economic, societal, and legal challenges to many geo-energy and waste-disposal projects. Existing tools to assess such problems are still inherently heuristic and mostly based on expert elicitation (so-called clinical judgment). We propose, as a complementary approach, an adaptive traffic light system (ATLS) that is function of a statistical model of induced seismicity. It offers an actuarial judgement of the risk, which is based on a mapping between earthquake magnitude and risk. Using data from six underground reservoir stimulation experiments, mostly from Enhanced Geothermal Systems, we illustrate how such a data-driven adaptive forecasting system could guarantee a risk-based safety target. The proposed model, which includes a linear relationship between seismicity rate and flow rate, as well as a normal diffusion process for post-injection, is first confirmed to be representative of the data. Being integrable, the model yields a closed-form ATLS solution that is both transparent and robust. Although simulations verify that the safety target is consistently ensured when the ATLS is applied, the model from which simulations are generated is validated on a limited dataset, hence still requiring further tests in additional fluid injection environments.

## Introduction

A significant proportion of the world’s global energy production relies on subsurface resources, such as oil, gas and coal production, as well as geothermal energy. In addition, the deep underground is increasingly used for waste storage; typical examples are wastewater from fracking operations and CO_2_ sequestration. However, these technologies are not “risk-free,” as shown by the increased frequency of induced seismicity cases around the globe. Recent examples include induced seismicity related to fracking and wastewater disposal^1–3^, gas extraction^4^, gas storage^5^, CO_2_ sequestration^6^, and renewable geo-energy^7–9^. Although some jurisdictions have enforced the use of maximum magnitude thresholds to limit the induced seismicity risk^10^, most of these rules remain heuristic. In this article, we argue that quantitative risk assessment and mitigation strategies rather than trial-and-error methods, should be essential tools to guarantee safety for society. The approach we advocate, which should be seen as a proof-of-concept, allows for an informed risk-cost-benefit analysis involving all stakeholders^11^.

Traffic light systems (TLS) are commonly used to mitigate induced seismicity risk by modifying the fluid injection profile^1,10,12,13^. A TLS is based on a decision variable (earthquake magnitude, peak ground velocity, *etc*.) and a threshold value above which actions (e.g. stopping the injection or reducing production rates) must be taken. Currently, the definition of this threshold is based on expert judgment and regulations^10,12,13^. Here, we propose a data-driven adaptive TLS, termed ATLS, which aims to overcome the limitations of the traditional heuristic methods. Here, the assignment of a magnitude threshold is based on a quantitative risk assessment, subject to a safety criterion imposed by the authorities (e.g., fixed probabilities of unaccepted nuisance, damage or fatalities). As a consequence, the ATLS is an objective and statistically robust mitigation strategy, which facilitates a fair and transparent regulatory process. This approach is in line with the procedures common for most other technological risks, such as in the hydropower, nuclear or chemical industries^14^. Model-based forecasting and alerting are already advocated elsewhere, such as in hurricane data assimilation and forecasting^15^.

## Results

### Predictive hazard model

A predictive model lies at the heart of any risk assessment. In the case of induced seismicity, a wide range of statistical and physics-based models exists^16,17^. Undoubtedly, more work is needed to develop, calibrate and validate new models; however, we believe that the missing link is the use of such models for deriving and monitoring quantitative risk thresholds. Here, we use as example a simple and yet robust model that forecasts the piecewise induced seismicity temporal, here daily, rate λ(*t*, *m* ≥ *m*_0_; θ) as:

1 $$\lambda (t,m\ge {m}_{0};{\rm{\theta }})=\{\begin{array}{cc}{10}^{{a}_{fb}-b{m}_{0}}\dot{V}(t) & ;\,t\le {t}_{shut-in}\\ {10}^{{a}_{fb}-b{m}_{0}}\dot{V}({t}_{shut-in})\exp (-\frac{t-{t}_{shut-in}}{\tau }) & ;\,t > {t}_{shut-in}\end{array}$$

where $\dot{V}$(*t*) is the injection flow rate as a function of time *t* in m^3^/day, θ = [*b*, *a*_*fb*_, τ] a set of model parameters describing the underground characteristics (earthquake size ratio, activation feedback in m^−3^ and mean relaxation time in days, respectively), *m*_0_ the minimum magnitude cutoff, and *t*_*shut-in*_ the shut-in time, also in days. Both *a*_*fb*_ and *b* can also be functions of time *t* (see Decision variable section). In this model, the injection or operation phase is described by a linear relationship between λ(*t*, *m* ≥ *m*_0_) and $\dot{V}$(*t*) in line with previous studies^17–19^. It derives directly from the linear relationship between $\dot{V}$ and overpressure^17^, hence assuming no change of injectivity during any given stimulation. The post-injection phase is described by a pure exponential decay representative of a normal diffusion process^17^. Although the Modified Omori Law is sometimes used to describe post-injection seismicity^20^, reasons remain mostly historical^21,22^. The proposed alternative is verified to be consistent with the tested data (see Methods section) and preferred on analytical grounds, being directly integrable in contrast with the Modified Omori Law, which is conditional on parameter values and may require the definition of an *ad hoc* upper bound^22^. Finally, no maximum magnitude *M*_*max*_ is imposed. It follows from Eq. (1) that induced seismicity is characterized by both the injection profile $\dot{V}(t)$, and the underground feedback described by the three-parameter set θ. The main limitations of the proposed model are discussed in detail later on.

In contrast with complex fluid modelling^16^, the closed-form Eq. (1) can be computed on-the-fly; moreover, it includes the mean relaxation time, τ, hence taking into account the long-term underground feedback after shut-in. Finally, being integrable, it leads in turn to a closed-form ATLS, as demonstrated in the next section. Although the physical process governing the rate of induced seismicity is more complex than what is represented by Eq. (1), this rate model is proven to be valid in a Poissonian probabilistic setting (see Methods section). Moreover, the physical processes are either not still clear (in fact, there is not an unanimous consensus among scientists), or computationally expensive. Therefore, pragmatism imposes the use of statistical models until both an agreement is found on the physics of induced seismicity and computational time of complex physical modelling is reduced.

**Figure 1:**
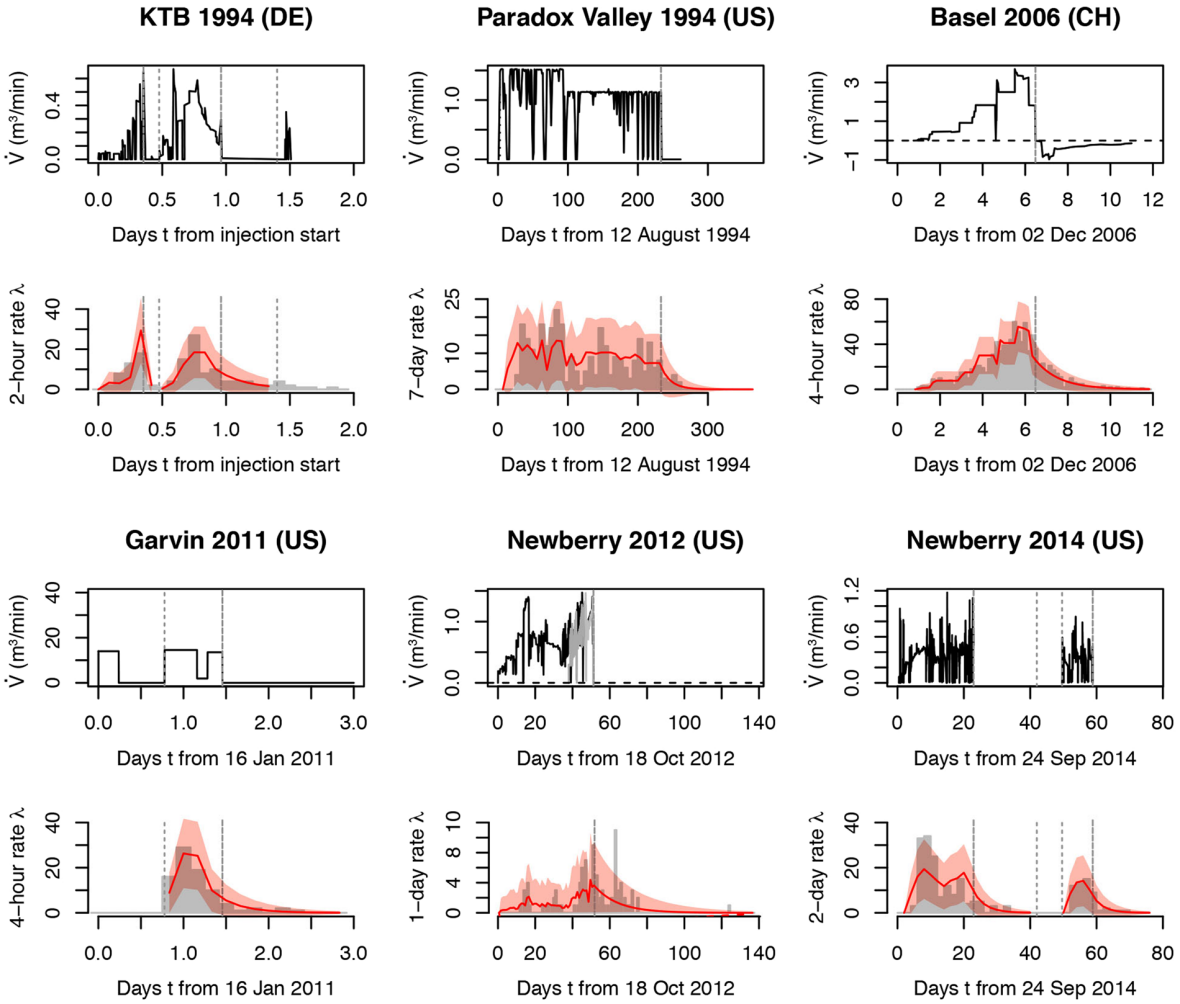
Induced seismicity model fitting of six fluid injection experiments: 1994 German Continental Deep Drilling Program (KTB); 1994 Paradox Valley, United States; 2006 Basel, Switzerland; 2011 Garvin, United States; and 2012–2014 Newberry, United States. For both KTB and 2014 Newberry, experiments are broken down into two separate stimulations, each with its own post-injection tail. The model (Eq. 1) is represented by the red curves on the induced seismicity time series with the ±3σ uncertainty envelope shown in light red. Dashed and dotted vertical lines indicate the shut-in times and sub-stimulation periods, respectively.

**Figure 2:**
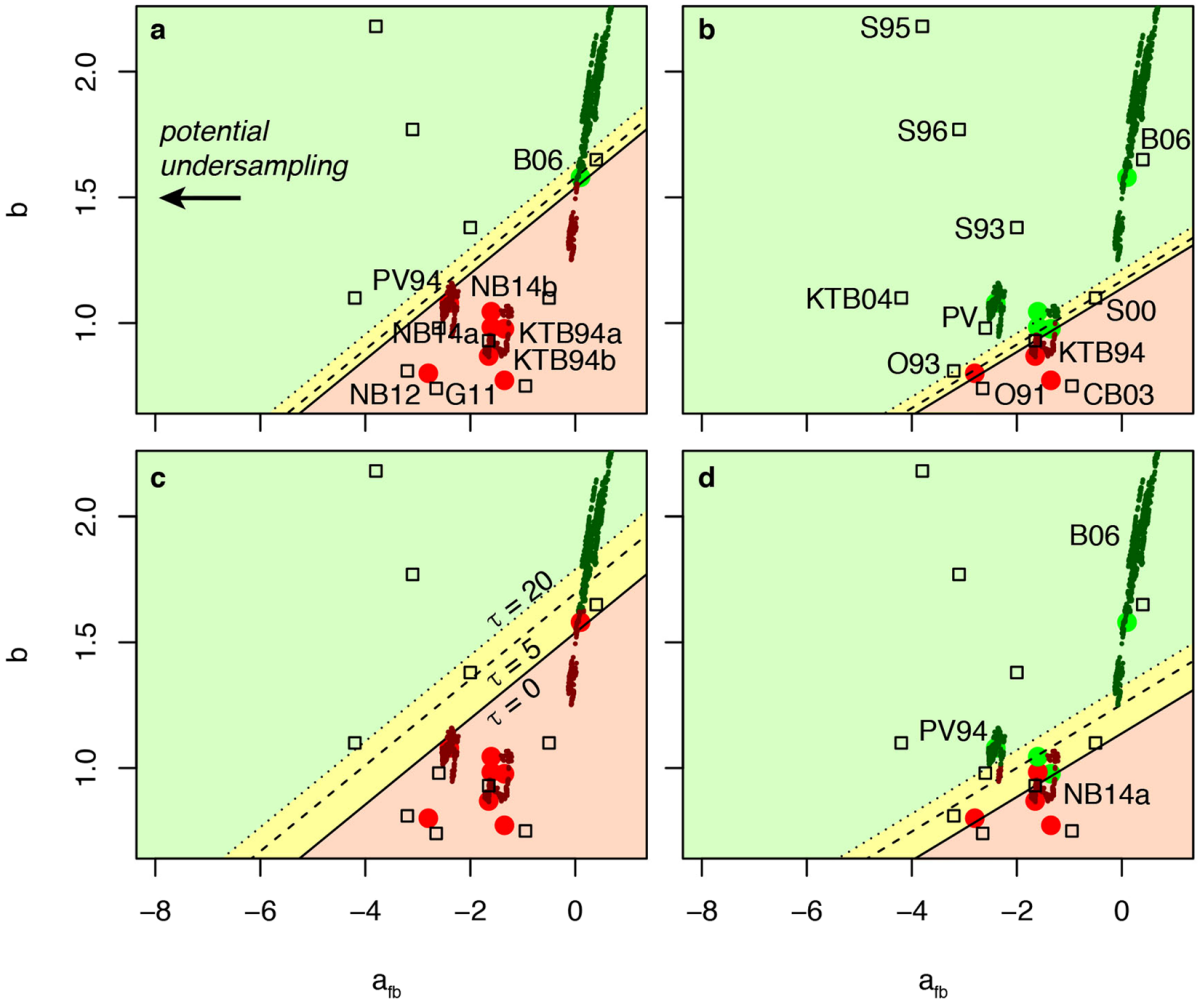
Three-parameter set θ = [b, a_fb_, τ] scattering in fluid injection experiments & impact on project validation for fixed safety threshold (*IR* ≤ 10^−6^) and different fluid injection scenarios. Dots represent θ estimates obtained in the present study (Fig. 1), squares the ones obtained by ref.^18^ (with no information on τ) and curves (made of successive dots) time-dependent estimates obtained where the earthquake catalogue is large enough (see Methods section). The hypothetical project injects a total volume V = 10,000 m^3^ of fluids with constant flow rate $\dot{{\rm{V}}}$ at a distance *d* from the nearest building: a. $\dot{{\rm{V}}}$ = 1 m^3^/min, *d* = 0 km; b. $\dot{{\rm{V}}}$ = 1 m^3^/min, *d* = 50 km; c. $\dot{{\rm{V}}}$ = 10 m^3^/min, *d* = 0 km and d. $\dot{{\rm{V}}}$ = 10 m^3^/min, *d* = 50 km. Lines represent the safety threshold for different values of τ (in days) (Eq. 2). Colour green or red indicates if the safety target is respected or not, respectively (darker colours are used in the case of time-dependent estimates). Letters represent sites: Basel (B), Cooper Basin (CB), Garvin (G), KTB, Newberry (NB), Ogachi (O), Paradox Valley (PV) and Soultz (S), followed by the experiment year’s last two digits.

The model (Eq. 1) was fitted to six induced seismicity sequences observed in fluid injection experiments from enhanced geothermal systems (EGS)^13,23–26^, the initial stage of a long-term brine sequestration^27^, and one fracking at an oil field^28^ (Table 1). The model succeeds to describe most of the data as shown in Figure 1 (see results of statistical tests in the Methods section – Note that the rare outliers above 3σ may be due to missing on-site data that may affect seismicity, such as unknown technical operations on wells, or to second-order physical processes missing in Eq. (1), as discussed below). The parameters are found to range over 0.8 ≤ *b* ≤ 1.6, −2.8 ≤ *a*_*fb*_ ≤ 0.1 m^−3^ and 0.2 ≤ τ ≤ 20 days and show a relatively large scattering between sites and between different stimulations at a same site (Table 2). It is important to note that we specifically chose those six datasets, as they had been made publicly available. Those cases are characterized by high pressures and flow rates, and are rich in induced earthquakes. This represents a selection bias and the *a*_*fb*_ parameter could decrease to much lower values elsewhere (Fig. 2). For instance, most injection wells in the U.S. do not cause felt earthquakes^29^; large *a*_*fb*_ variations between regions and sites might be explained by different regional crustal stresses^30^. The present sites provide however natural laboratories to test our model and the associated ATLS, without generalizing or inferring any high level of risk for all existing deep fluid injections. Figure 2 illustrates the parameters’ scattering, including results from past studies^18^ for EGS and other hard rock settings enlarging the range of values to 0.7 ≤ *b* ≤ 2.2 and −4.2 ≤ *a*_*fb*_ ≤ 0.4 m^−3^. It has been shown that in fracking environments, the activation feedback can be as low as *a*_*fb*_ = −9.25 m^−3^ ^18^.

**Table 1:** Source^*^ of stimulation experiment datasets.

Site (country ISO code), year	Injection profile	Earthquake catalog
Basel (CH), 2006	Digitized from (13)	(23)
Garvin (US), 2011	Digitized from (28)	(28)
KTB (DE), 1994	Digitized from (24)	(24)
Paradox Valley (US), 1994	http://www.usbr.gov/uc/wcao/progact/paradox/RI.html	http://www.usbr.gov/uc/wcao/progact/paradox/RI.html
Newberry (US), 2012	Digitized from (25)	http://fracture.lbl.gov/cgi-bin/Web_CatalogSearch.py
Newberry (US), 2014	Digitized from (26)	http://fracture.lbl.gov/cgi-bin/Web_CatalogSearch.py

^*^Online material last assessed in June 2017.

**Table 2:** Maximum likelihood estimates per stimulation experiment.

Experiment	*m*_0_	*b*	*a*_*fb*_ (m^−3^)	Σ^*^	τ (days)
B06	0.8	1.58	0.10	0.4	1.12
G11	1.0	0.77	−1.35	N/A	0.28
KTB94a	−1.5	0.98	−1.35	−1.65	0.03^†^
KTB94b	−1.4	0.87	−1.65	−1.65	0.22
PV94	0.6	1.08	−2.40	−2.6	14.13
NB12	0.2	0.80	−2.80	N/A	12.59
NB14a	0.0	0.98	−1.60	N/A	3.55
NB14b	0.2	1.05	−1.60	N/A	3.16

^*^Seismogenic index obtained by ref.^18^; ^†^unreliable, part of the tail being likely hidden by the KTB94b sequence.

### Safety criterion

A safety criterion is a probability of exceedance that can be fixed with respect to different safety metrics, such as fatalities, economic loss, building damage or level of nuisance^14^. Given the selected metric, the corresponding safety criterion can be converted in the magnitude space into the probability of exceedance Pr(*m* ≥ *m*_*saf*_) = *Y*, which will ensure that the acceptable level of risk is preserved at all time:

2 $${\rm{\Pr }}(m\ge {m}_{saf})=1-\exp \,\{-{10}^{{a}_{fb}-b{m}_{saf}}[V({t}_{shut-in})+\tau \dot{V}({t}_{shut-in})]\}=Y$$

with *m*_*saf*_ the magnitude at which the given safety limit (e.g., damage, fatality) is reached. Note that Eq. (2) is a closed-form expression where *V*(*t*) is the cumulative injected fluid volume, and *m*_*saf*_ and *Y* are derived from the safety criterion (see Methods section). Moreover, the set of parameters θ is updatable at any given time. The mapping from risk to earthquake magnitude is required to control injection operations based on short-term observations (see Decision Variable section). While peak ground velocity (PGV) is a more direct measure^12^, conversion to magnitude is in any case inevitable to estimate the risk potential of larger earthquakes from the *b*-value. It is also a unique measure, while PGV requires a location that is not trivial to assign. It should be added that no maximum magnitude *M*_*max*_ is imposed in Eqs (1–2). This implicitly assumes that both small to medium-size induced events and large triggered earthquakes on existing faults are treated the same way. This remains debated^31^ although a recent study^19^ demonstrated that the observed *M*_*max*_ in fluid injections is compatible with the null-hypothesis of the Gutenberg-Richter law with no upper limit. The role of *M*_*max*_ (and therefore of triggered earthquakes) is however only critical when the risk of fatalities (e.g., individual risk *IR*) is evaluated. For nuisance or minor damage thresholds, risk is more likely dominated by medium-size induced events. This important discussion has no significant impact on the method proposed, as proved in the Methods section.

Before an ATLS is set, the likelihood of failure of the planned project with respect to a specified limit state function defined by the safety criterion in magnitude space (Eq. 2) can approximately be determined. In this study, we select as main metric the annual individual risk (*IR*) over the entire project period, and as safety criterion *IR* ≤ 10^−6^ (i.e. the probability that a statistically representative individual dies for the introduced hazard), which is a threshold commonly enforced for hazardous installations^14^. Figure 2 shows the acceptable domain for a fixed limit state function, tested for different injection scenarios in which a hypothetical project plan is to inject a total volume *V* = 10,000 m^3^ of fluids at a depth of 4 km^32^. The injection profile is assumed to be flat with a constant flow rate $\dot{V}$ = 1 or 10 m^3^/min, having an impact on injection duration and tail behaviour (Eq. 2; Fig. 2). The project is considered to be located at a distance *d* = 0 km or 50 km from the nearest building. For a given site, with no knowledge of the underground feedback to fluid injection, project operators and regulators in an EGS setting (where high seismicity rates are common during stimulation) could use the known θ scattering for an *a priori* parameterization. This preliminary assessment shows the likelihood of the project to pass or fail the safety threshold. As shown in Figure 2, the results can be ambiguous, due to the large uncertainties associated with subsurface characteristics. Nevertheless, it provides a preliminary assessment of the risk reflecting the limited knowledge of the induced seismicity process. Future estimations of θ at additional injection sites will likely refine the results and improve the decision process. In addition, rules of decision-making under uncertainty can account for that ambiguity^9,33^. Decisions become more obvious in cases in which the diagram would be entirely green (clear go) or red (clear no-go). Underground stimulation activities in areas with low exposure (e.g. remote EGS plant locations with large distance *d* from the nearest habitations) would evidently have a lower induced-seismic risk and, thus, shrink the red area. The termination of the 2006 Basel EGS project was due to the high induced-seismic risk emerged from the high exposure of the urban built environment^7,9^. Note that Eq. (2) can be used to predetermine a distance *d* for which the induced-seismic risk would become acceptable—conditional to a given injection profile *V*(*t*) and parameter set θ (since *m*_*saf*_ is a function of *d*; see Methods section).

### Decision variable

The ATLS decision variable must be selected and updated as new data allows estimating θ more accurately, or if the planned injection scheme is changed. Here, a threshold earthquake magnitude *m*_*th*_ is used as decision variable. In particular, *m*_*th*_ is defined as the magnitude value for which mitigating actions must be taken, here corresponding to stopping injection, i.e.

3 $${m}_{th}=\frac{1}{b}\,{\mathrm{log}}_{10}[Y-{10}^{{a}_{fb}-b{m}_{saf}}\tau \dot{V}({t}_{shut-in})]+{m}_{saf}$$

(see Methods section). If *m*_*th*_ is updated “on-the-fly”, the project is guaranteed to meet the defined safety criterion.

To avoid reaching *m*_*th*_ before the planned stop of the fluid injection one may be inclined to reduce the flow rate $\dot{V}$, but here it would only delay the time at which the injection must stop, as the risk is mostly controlled by total volume injected *V* (Eq. 2; the secondary role of $\dot{V}$ is highlighted in Figure 2 by the change of width of the yellow band, for different $\dot{V}$ and τ). It is common practice to reduce $\dot{V}$ however^13^, but results of such action remain unclear^34^ since verification of a safety threshold requests a large number of experiments (see simulations below); indeed: (*i*) It is plausible that such action has no overall effect, the risk remaining the same in average over a fixed *V*; (*ii*) If such action has an effect, it would indicate that the model of Eq. (1) does not properly describe the role of different injection strategies. Despite the proposed model being classified as a pure statistical method, it is based on physical considerations. Its first term, for example, builds on the linear relationship between volume change and pressure^17^. Other relationships can be envisioned such as a bilinear relationship indicative of a change of injectivity^26^, which may explain some second-order relationship observed between flow rate and *M*_*max*_^35^. The linear relationship could also be shifted in time by including a minimum pressure threshold^17^ below which no induced seismicity is triggered. Adding such processes would likely allow for smarter mitigation strategies in which the shape of the injection profile would play a role. Since any model change would require the inclusion of additional parameters (which have yet to be constrained), and since Eq. (1) is verified to be consistent with most of the data tested, we consider Eq. (1) to be a reasonable first-order model for the proposed ATLS.

**Figure 3:**
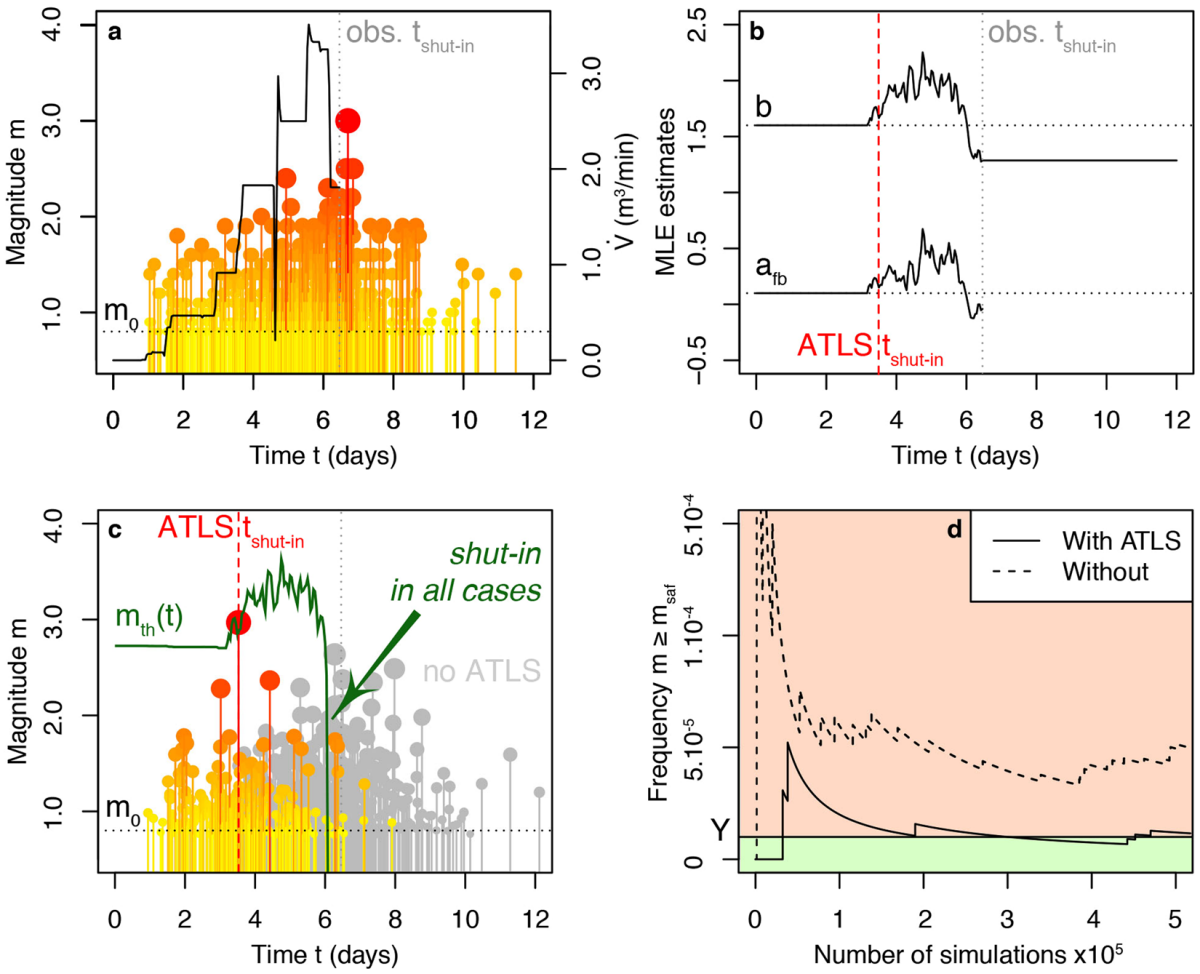
ATLS validation on synthetics of the 2006 Basel fluid injection experiment. (**a**) Observed time series and flow rate $\dot{V}(t)$ during the 2006 Basel experiment^13,23^; (**b**) Maximum likelihood estimates (MLE) of ground parameters *a*_*fb*_ and *b* over time determined from the Basel time series using a moving window of 100 events (see Methods section). Generic parameter values are here updated after the first 100 events are observed. Once injection stops at t_shut-in_, *b* is assumed to remain constant since no control on induced seismicity is possible after that time; (**c**) Simulated version of the 2006 Basel time series (in grey) and shorten (coloured) when injection is stopped by having an event with magnitude *m* > *m*_*th*_ (in green); (**d**) ATLS validation showing that the frequency of events with *m* ≥ *m*_*th*_ tends to *Y* over many simulations (fluctuations around *Y* represent the inherent uncertainty of the earthquake Poisson process, which decrease with increasing sample size).

To validate the ATLS in a realistic time-dependent setting, we simulate the Basel induced seismicity sequence using Eq. (1) with $\dot{V}\,$_Basel_(*t*) (Figs 1; 3a) and θ_Basel_(*t*) = [*b*(*t*), *a*_*fb*_(*t*), τ = 1.12 day] (Figs 2; 3b; Table 2). We use the safety threshold *IR* ≤ 10^−6^ with the nearest building above the borehole (*d* = 0 km), yielding *m*_*saf*_ = 5.8 (see Methods section). The value *m*_*th*_ as a function of time is shown in Figure 3c with two examples of induced seismicity time series. The first one, in grey, is a reproduction of the 2006 Basel experiment; the second one, coloured, is the case where the ATLS is used. Figure 3d finally shows that the safety target, while not respected for synthetic versions of the Basel experiment, is reached once the newly proposed ATLS (Eq. 3) is considered. Due to the stochastic nature of the earthquake process, the operational safety target is only reached on average over multiple sequences. It is plausible that an improved model with a non-linear relationship between flow rate and overpressure would open the possibility for mitigation strategies based on different injection profiles, hence potentially avoiding prematurely stopping the stimulation and the project.

## Discussion

The main purpose of this study was to present a statistical-based first order ATLS, which verifies that a quantitative safety criterion is ensured. An important benefit of the outlined ATLS approach to both operators and regulators is its transparency and execution speed (being a suite of simple closed-form expressions). By principle, its adoption would make any project in compliance with the safety threshold whatever the response of the underground. This does not ensure that a project is financially successful, but it gives the operator the maximum allowable chance to reach success, based on a quantitative risk-based method.

It is important to note that existing traffic light systems based on heuristics can already provide reasonable results (for instance, the magnitude threshold *m*_*th*_ = 2.9 fixed during the Basel experiment^13^ is not far off the *m*_*th*_(*t*) computed with the new ATLS; Fig. 3c). However, magnitude thresholds enforced by different jurisdictions vary significantly (with 0.5 ≤ *m*_*th*_ ≤ 4^10^) with no clear link with the standard risk-based safety criteria used in other hazardous industries^14^. Although the application of the ATLS in the decision process might appear at first complex, there is substantial evidence that the algorithmic (or actuarial) approach is superior to the so-called clinical approach of informal judgement^36,37^. This must apply too to induced seismicity prognostics, experts necessarily basing their judgements, consciously or intuitively, on past observations shown here to be reasonably well described by Eq. (1) (Fig. 1 and Methods section).

Finally, we suggest the following future directions: (1) improve the model (Eq. 1) by relating directly overpressure instead of flow rate to induced seismicity. Due to potential changes in injectivity, gas kicks and other processes, overpressure is likely to provide a better proxy than injected fluid volume; (2) test the statistical model in other fluid injection environments; (3) improve the updating of the parameter space by using a hierarchical Bayesian framework where the uncertainties of the model parameters are taken into account; and (4) modify the mapping from risk to magnitude space for site-specific conditions which likely vary between fluid injection locations. The present study demonstrated the power of the actuarial approach and should be considered as a proof-of-concept for future physics-based induced seismicity models, more sophisticated engineer-based risk assessments, and improved mitigation strategies.

## Methods

### Time series analysis

The induced seismicity temporal rate model λ(*t*) of Eq. (1) is fitted to the induced seismicity data by using the maximum likelihood estimation (MLE) method^38^. The probability *p*_*i*_ that the observed number *n*_*i*_ of induced earthquakes results from a Poisson process^39^ with rate λ_*i*_ is

4 $${p}_{i}=\frac{{\lambda }_{i}^{{n}_{i}}\exp (-{\lambda }_{i})}{{n}_{i}!}$$

which yields the log-likelihood function

5 $$LL(\theta ,X)=\sum _{i=1}^{imax}\,\mathrm{ln}({p}_{i})=\sum _{i}^{imax}[{n}_{i}\,\mathrm{ln}({\lambda }_{i})-{\lambda }_{i}-\,\mathrm{ln}({n}_{i}!)]$$

where *X* = {*n*_1_, …, *n*_*i*_, …, *n*_*imax*_} is the observation set and θ = [*a*_*fb*_, *b*, τ] is the parameter set of Eq. (1). The maximum likelihood estimate of θ is finally θ_MLE_ = arg max_θ_ *LL*(θ, *X*). *m*_0_ is fixed to *M*_*c*_, the completeness magnitude defined as the magnitude bin with the highest number of events^40^. *b* is estimated independently of λ, also based on the MLE method^41^. The model is fitted to 8 datasets (from 6 stimulations in various injection settings; Table 1); the resulting maximum likelihood estimates are listed in Table 2.

### Sensitivity analysis

Temporal changes in *a*_*fb*_ and *b* are evaluated for induced seismicity sequences that are large enough (i.e., made of hundreds of events, such as in 1994 Paradox Valley, 2006 Basel and 2014 Newberry). The parameters are estimated using a moving window with constant event number *n* = 100. Before *n* is reached, MLE estimates obtained in retrospect (Table 2) are used, as shown in Figure 3b. In a prospective case, generic values should be used, e.g. the median or mean of values taken by the parameters in previous experiments. Since the post-injection phase is not considered in the sensitivity analysis, *a*_*fb*_ is directly obtained from Eq. (1) so that

6 $${a}_{fb,i}=b{m}_{0}+{\mathrm{log}}_{10}\,n-{\mathrm{log}}_{10}({V}_{i+1}-{V}_{i})$$

with *i* the incremental window step. Noteworthy, *n* ≥ 100 is reached only for high *a*_*fb*_ and/or low completeness *M*_*c*_. Depending on the underground *a priori* knowledge, the injection profile plan, and the safety criterion (Eq. 3), one may estimate what a reasonable *M*_*c*_ would be for parameter updating over time. This may require further seismic network planning, with the number and spatial arrangement of seismic stations potentially derived from the simple function ${M}_{c}(d,k)={c}_{1}d{(k)}^{{c}_{2}}+{c}_{3}$, where *d* is the distance to the *k* ^th^ nearest seismic station and *c*_1_, *c*_2_ and *c*_3_ empirical parameters^42^ (although there are additional theoretical limits on event detection that must be taken into account^43^).

### Post-injection data analysis

While the linear relationship between λ and $\dot{V}$ is well established^17–19^, the pure exponential behaviour of the induced seismicity post-injection tail has only been demonstrated for the 2006 Basel case^17^. Here, we compare three relaxation models: pure power law $\lambda (t)\propto {t}^{-\alpha }$, pure exponential $\lambda (t)\propto \exp (-t/\tau )$ and stretched exponential $\lambda (t)\propto {t}^{\beta -1}\exp (-{(t/\tau )}^{\beta })$ by using the Akaike Information Criterion^44,45^. We find that, of the 7 datasets (discarding KTB94a, which tail is likely cut), 5 are best described by the pure exponential function. The 2 others are best described by a stretched exponential function with stretching parameter β = 0.9 and 0.7, for PV94 and NB14b, respectively (β = 1 representing the pure exponential). This justifies the use of a pure exponential in Eq. (1) hence limiting θ to a simple three-parameter set.

### Model goodness-of-fit

Figure 1 shows the general agreement between model and data by visual inspection. Additionally we test Eq. (1) against our datasets using the Kolmogorov-Smirnov (KS) confidence bounds^46^. We first convert the dataset ${{\mathscr{D}}}^{(t)}$ into a transformed dataset ${\tilde{{\mathscr{D}}}}^{({\rm{{\rm T}}})}$ as follows:

7 $$\{\begin{array}{c}{{\mathscr{D}}}^{(t)}=[{t}_{1},\ldots ,{t}_{i},\ldots ,{t}_{n}]\to {\tilde{{\mathscr{D}}}}^{({\rm{{\rm T}}})}=[{{\rm{{\rm T}}}}_{1},\ldots ,{{\rm{{\rm T}}}}_{i},\ldots ,{{\rm{{\rm T}}}}_{n}]\\ {{\rm{{\rm T}}}}_{i}={\int }_{0}^{{t}_{i}}\lambda (t,\theta )dt\end{array}$$

**Figure 4:**
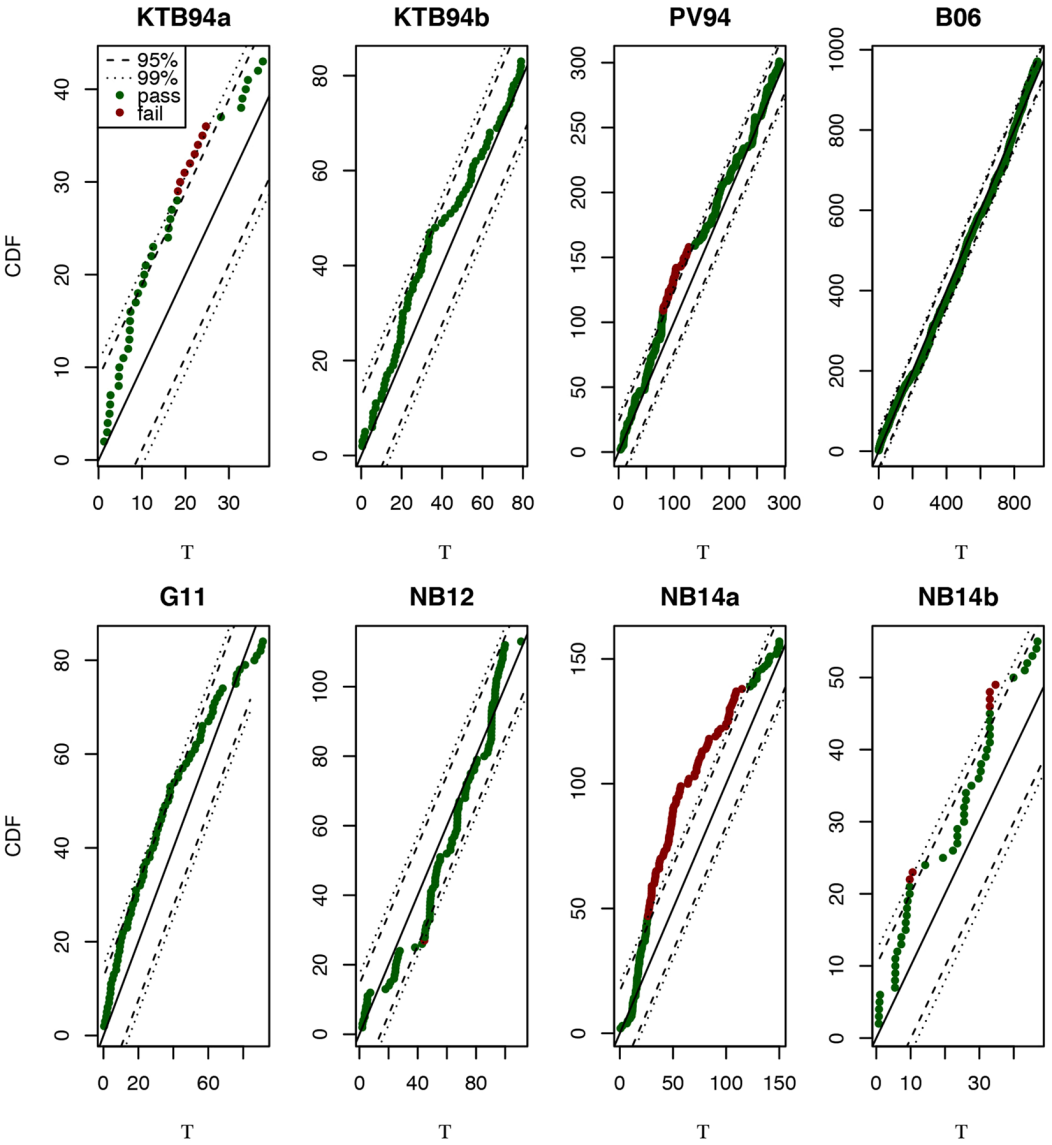
Goodness-of-fit of the model represented by Eq. (1). Using the Kolmogorov-Smirnov (KS) confidence bounds, we find that the model performs well for the datasets KTB94b, B06 and G11; fairly well for the datasets KTB94a, PV94, NB12 and NB14b; and poorly for dataset NB14a (see the Methods section for details).

The two datasets are equivalent but with the distribution of ${\tilde{{\mathscr{D}}}}^{({\rm{{\rm T}}})}$ being the one of a uniform Poisson process with unit rate. Then using the KS confidence bounds, we estimate graphically whether the empirical cumulative distribution function (CDF) of ${\tilde{{\mathscr{D}}}}^{({\rm{{\rm T}}})}$, ${F}_{\tilde{{\mathscr{D}}}}({\rm{{\rm T}}})$, deviates significantly from the CDF of the uniform distribution, *F*_*U*_(Τ). Here, we use the confidence bounds not to perform a KS statistical test but rather to examine the physical-engineering evidence against the proposed model. When ${F}_{\tilde{{\mathscr{D}}}}({\rm{{\rm T}}})$ is within the 95–99% KS bounds, we classify the model as performing well; when ${F}_{\tilde{{\mathscr{D}}}}({\rm{{\rm T}}})$ falls locally outside the 95–99% KS bounds, we classify the model as performing fairly well; when ${F}_{\tilde{{\mathscr{D}}}}({\rm{{\rm T}}})$ falls extensively outside the 95–99% KS bounds, we classify the model as performing poorly. Results are shown in Figure 4 with the dashed lines representing the two-sided 95% and 99% confidence intervals. The following conclusions can be drawn: The model performs well for the datasets KTB94b, B06 and G11; fairly well for the datasets KTB94a, PV94, NB12 and NB14b; and poorly for dataset NB14a. Given the range of different datasets, we conclude that the rate model expressed in Eq. (1) describes fairly well the relationship between injection flow rate and fluid-induced seismicity rate.

### Time series simulation

We simulate induced seismicity time series with time-dependent flow rates $\dot{V}$(*t*) and θ(*t*) = [*a*_*fb*_(*t*), *b*(*t*), τ] by using the thinning method for the injection phase^47^. For the post-injection phase for which τ is assumed constant, as no control over post-injection seismicity is possible, the inversion method is used instead to simulate event occurrence times *t* ^48^. Magnitudes *m* are also simulated using the inversion method (for both injection and post-injection phases with *b* time-dependent in the first phase).

### Risk-to-magnitude mapping

We translate the safety threshold *IR* = 10^−6^ as Pr(*I* = 9) = 10^−5^ assuming that a statistically average “poor” building collapse is reached for a ground intensity *I* = 9 (i.e., most buildings of class A in the EMS98 code suffer collapse)^49^ and that once a building collapses, there is a 10% chance of individual fatality^50^. The magnitude *m*_*saf*_ is then estimated from an intensity prediction equation (IPE). For induced seismicity, we use the IPE derived from the U.S. Geological Survey “Did You Feel It?” rich database^51^, corrected for induced seismicity^52^:

8 $$I({d}_{hyp})={c}_{1}+{c}_{2}({m}_{tecto}-6)+{c}_{3}{({m}_{tecto}-6)}^{2}+{c}_{4}{\mathrm{log}}_{10}\,{d}_{hyp}+{c}_{5}{d}_{hyp}+{c}_{6}{m}_{tecto}{\mathrm{log}}_{10}\,{d}_{hyp}+3\sigma $$

with *m*_*tecto*_ the earthquake magnitude in the tectonic context, ${d}_{hyp}=\sqrt{{d}^{2}+{h}^{2}}$ the hypocentral distance in km (point source hypothesis at depth *h* = 4 km), *c*_1_ = 11.72, *c*_2_ = 2.36, *c*_3_ = 0.1155, *c*_4_ = −0.44, *c*_5_ = −0.002044, *c*_6_ = −0.479 (*38*), σ = 0.4, *m*_*saf*_ = *m*_*tecto*_ + *m*_*corr*_ and *m*_*corr*_ = 0.82. The correction is based on the observation that induced earthquakes would seem less severe in average than tectonic ones. We assume that the Modified Mercalli Intensity (MMI)^49^ and the USGS Community Internet Intensity (CII)^51,52^ are equivalent for sake of simplicity. For a specific project, a fully probabilistic risk approach^9^ is recommended to derive the parameters *Y* and *m*_*saf*_ of the ATLS closed-form expressions (Eqs 2–3). Here, the safety thresholds shown in Figure 2 use *Y* = 10^−5^ with *m*_*saf*_(*d = *0 km) = 5.8 and *m*_*saf*_(*d = *50 km) = 7.9 (equivalent in the tectonic case to 5.0 and 7.1, respectively). This simplified approach is used to illustrate our ATLS proof-of-concept for a general case with no site-specific conditions. A detailed risk analysis, which would integrate risk over the full magnitude range^9^, remains outside the scope of the present study. The high values of *m*_*saf*_ are due to using *IR* as the safety metric. Some experts may disagree that such high magnitudes can be reached in the induced seismicity context^31^, although it is statistically plausible^19^. The choice of the safety metric has however no impact on the validity of the proposed method. For example, using the minor damage threshold Pr(*I* = 6) = *Y*_2_ (i.e., most buildings of class A in the EMS98 code suffer negligible to slight damage) would yield *m*_*saf*2_(*d* = 0 km) = 4.0. From Eq. (3), the same ATLS *m*_*th*_ would be computed for both Pr(*I* = 9) = 10^−5^ and Pr(*I* = 6) ≈ 10^−3.1^ assuming e.g. *b* = 1 in log_10_(*Y*_2_) = log_10_(*Y*) + *b*(*m*_*saf*_ − *m*_*saf*2_).

### Decision variable definition

The threshold *m*_*saf*_ is fixed such that Pr(*m* ≥ *m*_*saf*_) = *Y*. Assuming that earthquakes follow a non-homogeneous Poisson process,

9 $${\rm{\Pr }}(m\ge {m}_{saf})=1-\exp [-{\rm{\Lambda }}(T,m\ge {m}_{saf})]=Y$$

where ${\rm{\Lambda }}(T,m\ge {m}_{saf})={\int }_{0}^{T}\lambda (t,m\ge {m}_{saf};{\rm{\theta }})dt$ is the mean cumulative number of events and *T* is the observation time. For sufficiently large *T* (i.e. *T* >>  *t*_*shut-in*_ + τ),

10 $${\rm{\Lambda }}(T,m\ge {m}_{saf})={10}^{{a}_{fb}-b{m}_{saf}}[V({t}_{shut-in})+\tau \dot{V}({t}_{shut-in})]$$

with *V*(*t*) the cumulative injected volume and Λ(*T*, *m* ≥ *m*_*saf*_) ≈ *Y* for Pr(*m* ≥ *m*_*saf*_) << 1. Eq. (2) is then obtained by injecting Eq. (10) into Eq. (9) (note that one could replace *Y* by –ln(1-*Y*) in Eq. (2) with an impact on the results only if *Y* was tending to 1, which is unlikely in safety norms). We then define the ATLS as the operational magnitude threshold *m*_*th*_ at which the injection is stopped in order to meet the safety target. Note that *m*_*th*_ also provides the completeness magnitude to attain in the region and thus a basis for seismic network monitoring planning^42,53^. We thus obtain the following system of equations (for *Y* << 1):

11 $$\{\begin{array}{c}{10}^{{a}_{fb-b{m}_{saf}}}[V({t}_{shut-in})+\tau \dot{V}({t}_{shut-in})]\approx Y\\ {10}^{{a}_{fb}-b{m}_{th}}V({t}_{shut-in})=1\end{array}$$

The second equation is always true since the expected number of events with *m* ≥ *m*_*th*_ is one, given the assumption that injection is stopped for *t* = *t*_*shut-in*_ as soon as *m* ≥ *m*_*th*_ is first observed. Substituting *V*(*t*_*shut-in*_) in the first equation of this system yields Eq. (3). It is worth noting that omitting the post-injection tail effect with τ = 0 yields the basic frequency-magnitude distribution threshold *m*_*th*_ = log_10_(*Y*)/*b* + *m*_*saf*_.

### Data availability

All the data used in this study are publicly available. For more information, please contact arnaud.mignan@sed.ethz.ch.
